# Erythromycin leads to differential protein expression through differences in electrostatic and dispersion interactions with nascent proteins

**DOI:** 10.1038/s41598-018-24344-9

**Published:** 2018-04-24

**Authors:** Hoang Linh Nguyen, Dang Lan Pham, Edward P. O’Brien, Mai Suan Li

**Affiliations:** 1 Institute for Computational Sciences and Technology, Ho Chi Minh City, Vietnam; 2 Department of Chemistry, Pennsylvania State University, University Park, Pennsylvania, 16802 USA; 3 Institute of Physics, Polish Academy of Sciences, Al. Lotnikow 32/46, 02-668 Warsaw, Poland

## Abstract

The antibiotic activity of erythromycin, which reversibly binds to a site within the bacterial ribosome exit tunnel, against many gram positive microorganisms indicates that it effectively inhibits the production of proteins. Similar to other macrolides, the activity of erythromycin is far from universal, as some peptides can bypass the macrolide-obstructed exit tunnel and become partially or fully synthesized. It is unclear why, at the molecular level, some proteins can be synthesized while others cannot. Here, we use steered molecular dynamics simulations to examine how erythromycin inhibits synthesis of the peptide ErmCL but not the peptide H-NS. By pulling these peptides through the exit tunnel of the *E.coli* ribosome with and without erythromycin present, we find that erythromycin directly interacts with both nascent peptides, but the force required for ErmCL to bypass erythromycin is greater than that of H-NS. The largest forces arise three to six residues from their N-terminus as they start to bypass Erythromycin. Decomposing the interaction energies between erythromycin and the peptides at this point, we find that there are stronger electrostatic and dispersion interactions with the more C-terminal residues of ErmCL than with H-NS. These results suggest that erythromycin slows or stalls synthesis of ErmCL compared to H-NS due to stronger interactions with particular residue positions along the nascent protein.

## Introduction

Antibiotics are a type of antimicrobial that kills bacteria by inhibiting their growth and replication. Antibiotics target a diverse range of molecules and processes including DNA gyrase, RNA polymerase, cell wall synthesis, metabolic pathways and protein synthesis^1^. Due to the central nature of protein synthesis to life, many different antibiotics target and disrupt this process. Protein production occurs via the action of the ribosome, which covalently links amino acids together through the catalytic action of the peptidyl transferase center (PTC). Newly synthesized protein segments leave the ribosome through the exit tunnel^2^, a 10 nm long, irregularly-shaped tunnel that is on average 1.5 nm in diameter. The passage of a newly synthesized protein through the exit tunnel can be dramatically slowed due to interactions between the exit tunnel wall and sequence motifs within the primary structure of nascent proteins^3–5^. Additionally, the binding of ligands, including antibiotics, in the exit tunnel can also inhibit protein synthesis^1,4,6,7^.

The strong activity of ribosome-targeting macrolides against many gram-positive microorganisms indicates that they effectively inhibit protein production. Like the antibiotics chloramphenicol and puromycin, macrolides stop protein synthesis in the elongation phase of translation. However, instead of directly binding to the PTC and disrupting its structure, macrolides bind in the exit tunnel one or more nanometers from the PTC^1,8^. These macrolides decrease the diameter of the exit tunnel, leading to steric clashes with nascent peptides^8^. For instance, the ^64^KVE^62^ segment of the MarR peptide clashes with the cladinose and lactone moieties of both erythromycin (ERY) and Azithromycin (AZ) causing ribosome stalling^8^. In the case of the EngD peptide, only AZ induces ribosome stalling while ERY does not. The mechanism of ribosome stalling by macrolides is not limited to a narrowing of the exit tunnel. Vázquez-Laslop and coworkers^6^ showed that when the cladinose sugar group of ERY is replaced by substituents that are as bulky as cladinose, the resulting macrolide cannot stall protein synthesis. While Vázquez-Laslop^9^, Johansson^10^, and Arenz^11^ and their coworkers observed that mutating amino acids in the region ^6^IFVI^9^ of the peptide ErmCL can relieve stalling and allow synthesis of the nascent protein. These results demonstrate that the ability of macrolides to inhibit protein synthesis involves a combination of excluded volume interactions and attractive inter-molecular interactions between the nascent protein and antibiotic.

Although macrolides do not directly contact the PTC, the interaction between the peptide and macrolide can alter the PTC’s structure. By hindering the movement of the peptide through the exit tunnel, the nascent chain can compress and push back on the P-site, altering the structure of nucleotides within the PTC and thereby suppress catalysis of peptide bond formation. For example, in the presence of ERY, rRNA nucleotide U2585’s position is shifted within the tunnel, which could sterically conflict with the ErmCL nascent chain^11^. Ramu and coworkers^12^ demonstrated that the presence of ERY and ErmAL1 peptide in the exit tunnel alters the properties of the A-site, preventing particular tRNA’s from binding. The binding of macrolides in the exit tunnel can even alter the conformation of the PTC before the sequence of peptide has reached the macrolide through an allosteric mechanism^13^.

The exit tunnel can act synergistically with macrolides to inhibit protein synthesis, and altering the ribosomal nucleotides and residues that line the tunnel can lead to macrolide-resistant bacteria^3,5,6,14,15^. Mutations of the 23S ribosomal RNA (a component of the 50S ribosomal subunit) can confer macrolide resistance^15^. The mutations at A2058 and A2059, which are located on the surface of the tunnel mediate ERY resistance in multiple species^16,17^. The C2610U mutation in *Escherichia coli*’s (*E. coli*) ribosome reduces ERY-dependent ribosome stalling of ErmCL peptide twofold^6^, implying that the interaction between the exit tunnel and macrolide plays an important role in inhibition. Mutations in other components of 50S subunit can also contribute to macrolide resistance^18^. The mutation Δ^82^MKR^84^ in ribosomal protein L22, for example, confers antibiotic resistance in *E. coli*, *Thermus thermophilus* and *Haloarcula marismortui*^19^. Distinct amino acids and peptides attached to macrolide derivatives can establish interactions with components of the ribosomal tunnel that enhance the ribosome binding and inhibitory properties of macrolides^20^. For example, attaching Ala-Ala to the C20 aldehyde position of tylosin prevents covalent bonding between tylosin and A2062 of the ribosomal tunnel, but establishes a new interaction with the tunnel that improves inhibitory properties of tylosin.

A fascinating characteristic of macrolides is that even when they can tightly bind to wild-type bacterial ribosomes, they cannot block the synthesis of all proteins. Kannan and coworkers^14^ have identified protein sequences in *E. coli* and *S. aureas* that can bypass the drug-obstructed tunnel to become partially or fully synthesized. A key finding is that the ability to bypass macrolides does not depend on the entire sequence of the synthesized protein, but rather on its N-terminal sequence^14^. The N-terminal segment of H-NS can be synthesized even when erythromycin is bound in the exit tunnel, although its movement is slowed down^10,14,21^. Furthermore, while the synthesis of a protein known as OsmC is inhibited by erythromycin, this inhibition is overcome when various lengths of H-NS’s N-terminal residues are covalently attached to the N-terminus of OsmC, saturating at 12 H-NS residues^14^. Erythromycin is still bound in the tunnel after translation of the entire H-NS protein^14^, indicating that H-NS peptide bypasses erythromycin. Despite the important role of macrolide resistance in antibiotic development, motifs of peptides that are stalled by erythromycin, telithromycin and azithromycin have been extensively studied^13,21^ while the identification of motifs that can bypass macrolides has received less focus.

Here, we examine the molecular interactions that allow H-NS to bypass erythromycin and prevent ErmCL from progressing through the exit tunnel using steered molecular dynamics (SMD) simulations. In SMD simulations, segments from these two proteins are pulled along the ribosome exit tunnel using an artificially imposed force at a constant velocity in the presence and absence of ERY. We find that while ERY makes it harder to pull either peptide through the tunnel exit, a much larger force and work is required to move ErmCL compared to H-NS. Decomposing the energetic components, we find the interaction energy of ErmCL with ERY is more favorable than that of H-NS. These results indicate that the differential impact of ERY on these two protein’s arises from stronger intermolecular interactions between ERY and C-terminal residues of ErmCL than H-NS.

## Materials and Methods

### Starting structures

The structure of ERY bound to the *E. coli* ribosome was obtained from protein data bank (PDB) file with id 4V7U. Because this system is very large, ribosomal residues and bases were deleted that were more than 40 Å away from the center of mass of ERY. Initial structures of ErmCL (sequence: MGIFSIFVI) and H-NS (MSEALKILNNIR) peptides off of the ribosome were created using the RaptorX webserver^22^ and equilibrated in bulk solution using molecular dynamics (MD) simulations. Specifically, the peptides were solvated in a cubic box with TIP3P water molecules and the AMBER99SB-ILDN force field was utilized. GROMACS version 5.1.2^23^ was used to perform the simulations. The structures were equilibrated for 200 ps in the NVT ensemble followed by 5 ns in the NPT ensemble at 330 K and 1 bar. The system was then simulated for another 100 ns per trajectory at 330 K in the NPT ensemble. Peptide structures that had no secondary structure were identified in the simulations and selected for later insertion into the ribosome exit tunnel. A ribosome without ERY was created by deleting ERY from the ribosome-ERY crystal structure.

**Figure 1:**
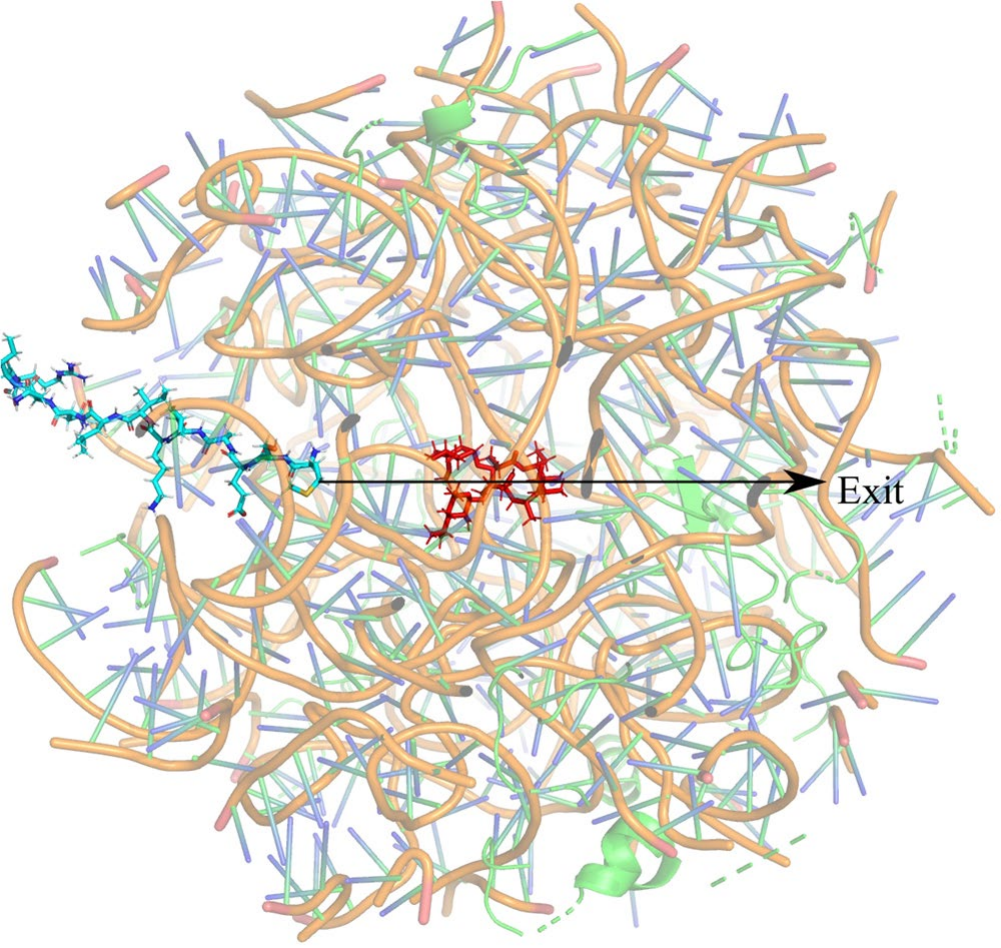
An illustration of a starting simulation structure. The ribosome (transparent cartoon), ERY (red), and nascent chain (cyan) are shown. The black arrow indicates the pulling direction applied to the N-terminal residue of the nascent chain in the SMD simulations. The arrow also lies along the long axis of the ribosome exit tunnel. “Exit” denotes the exit side of the tunnel.

To insert the ErmCL and H-NS peptides into the truncated ribosome we used a home-made program based on an optimization algorithm named Differential Evolution^24^. The input parameters for this program were set to a scaling factor F = 0.5 and crossover probability of P_c_ = 0.9, and were chosen based on trial and error attempts. The space occupied by the nascent peptide was divided into 60 × 60 × 60 discrete mesh points and the objective function defined as the number of mesh points that overlap with the ribosome. We used the additional constraint that the N-termini of the peptides be located near the base 2602 of the ribosome. As expected, after insertion the N-termini of the peptides was found to be closer to ERY than their C-termini (Fig. 1). The exit tunnel was aligned along the z-axis of the local coordinate system. The ribosome-peptide systems were then neutralized by adding 129 Mg^2+^ and 186 Na^+^ ions. Na^+^ ions were used instead of K^+^ ions because K^+^ and Cl^-^ can erroneously form salt crystals when using the AMBER force field. We then performed MD simulations in the gas phase for 2 × 10^5^ integration steps, which allowed the ions to rapidly diffuse to their binding sites on the ribosome. The heavy atoms of the peptide, ribosome and ERY were harmonically restrained during these simulations. The system was then solvated with TIP3P water molecules with an additional 0.1 M Na^+^ and Cl- ions. In total, about 30,000 water molecules were used in the simulations.

### SMD simulations

Following the conventional SMD approach^25^, the truncated ribosome was split into three regions. Bases and residues containing one or more atoms within 28 Å of ERY comprised the dynamic region. The buffer region is between 28 and 34 Å, while the remaining portion belongs to the outer region. Atoms within the dynamic region were not subject to any restraints during the SMD simulation course, but harmonic restraints were applied to non-hydrogen atoms within the buffer and outer regions with spring constants of 5 and 10 kcal mol^−1^ Å^−2^, respectively. The ribosomes were solvated in a rectangular box with distances between the system and box edge of 12 Å, 12 Å and 28 Å along the $x$, $y$ and $z$ axes, respectively. The AMBER99SB-ILDN force field was used in the SMD simulations, whereas force-field parameters for ERY were taken from the work of Solthiselvam and coworkers^13^. The constant velocity and the cantilever spring constant were set to 5 m/s in the $z$-direction and 1,000 kJ mol^−1^ nm^−2^. The cantilever was connected to the center of mass of the N-terminal residue of the nascent peptide. Because there is no experimental evidence that ERY diffuses out the tunnel during protein synthesis, a harmonic restraint, with a force constant of 1000 kJ mol^−1^ nm^−2^, was applied to the heavy atom closest to the center of mass of ERY. The PME method^26^ was applied to calculate electrostatic interactions using a 1 nm cutoff. We used only one starting structure for each peptide. In each SMD run, the system was first minimized by the steepest descent algorithm and equilibrated in the NVT^27^ ensemble for 200 ps and then in the NPT^28^ ensemble for 10 ns. The final structures from this NPT equilibration were used as starting structures for the production SMD simulations. A total of 30 and 34 SMD trajectories were performed for ErmCL and H-NS, respectively.

### Mimicking nascent peptide elongation

In order to mimic the real situation in which a new amino acid is added to a nascent peptide we first generated five different starting structures of the nascent peptide using the Differential Evolution program. At the start of the simulation, only the non-bonded parameters of the first nascent peptide residue was turned on. Then, once the nascent peptide had been pulled a distance of 4 Å into the tunnel, the non-bonded interactions of the next residue were turned on. The structure of the system with the newly added residue was minimized by the steepest descent algorithm and equilibrated in the NVT ensemble for 20 ps and then in the NPT ensemble for 100 ps. This procedure was repeated until the interactions of the most C-terminal residue were turned on. After this, we continued the SMD simulations. A total of 10 trajectories were performed in this manner for ErmCL and H-NS.

### Definitions of computed quantities

A hydrogen bond is considered to be formed when the distance between a donor atom and acceptor is within 3.5 Å and the angle of the acceptor, hydrogen and donor is greater than or equal to 135 degrees. A contact between bases of the ribosome and residues of the nascent peptide is present when the distance between their respective centers of mass is less than 6.5 Å. The average non-bonded interaction force at a time point is calculated as follows:

$${\langle {F}_{I}\rangle }_{J}=\frac{1}{{N}_{traj}}\sum _{n=1}^{{N}_{traj}}{|\sum _{j}\overrightarrow{{F}_{IJ}}|}_{n}$$

${\langle {F}_{I}\rangle }_{J}$ is the average of the magnitude of the force for all trajectories that system *J* acts on system $I$. $\sum _{j}\overrightarrow{{F}_{IJ}}$ is the force that system *J* acts on system *I* of one trajectory. $\overrightarrow{{F}_{IJ}}=\sum _{i}\overrightarrow{{F}_{ij}},$ where $\overrightarrow{{F}_{ij}}={\overrightarrow{F}}_{electrostatic}(i,j)+{\overrightarrow{F}}_{vanderWaals}(i,j)\,\,$is the force that atom *j* of system *J* acts on all atoms *i* of system *I*. The self non-bonded interaction force is the total force, per residue, acted on by the others. The charges and van der Waals parameters of the atoms for calculating forces are the same as in the MD simulation. The work associated with pulling the nascent chain through the tunnel was calculated by the Trapezoid Rule $W={\int }^{}Fdx\approx \sum _{i=1}^{N}({F}_{i+1}+{F}_{i})({x}_{i+1}-{x}_{i})/2,$ where *i* represents simulation step, *F* is the pulling force and *x* is the pulling coordinate.

## Results

### In the absence of ERY, H-NS is harder to translocate through the tunnel than ErmCL

We used SMD simulations to calculate the pulling force versus time profiles of pulling ErmCL and H-NS through the exit tunnel in the absence of ERY (Figure S1). ErmCL has a mean rupture force, denoted *F*_max_, of 468.6 pN (95% CI: [442.6, 494.6]), which is lower than the 599.9 pN (95% CI: [585.2, 614.6]) value associated with H-NS (Table 1). This result is perhaps surprising because in the presence of ERY, ErmCL’s synthesis is stalled but not that of H-NS^14^. The distribution of rupture forces of ErmCL are statistically different from those of H-NS (Student’s t-test, *p*-value 0.007). Integrating the force profiles in Figure S1 yields the non-equilibrium work, *W*_pull_, of translating these peptides whose values are 305 and 507 kcal/mol for ErmCL and H-NS, respectively (Table 2). Therefore, there is a statistically significant difference between the distributions of *F*_max_ and *W*_pull_ for these two peptides, indicating that H-NS interacts more strongly with the ribosome exit tunnel than ErmCL. Nevertheless, in the absence of ERY both peptides are completely synthesized^10^.

**Table 1:** Rupture force in the absence and presence of ERY in the exit tunnel.

Rupture force (pN)	ErmCL	H-NS
Without ERY	468.6 ± 26.0	599.9 ± 14.7
With ERY	2639.9 ± 119.8	1394.3 ± 88.9

Error bars represent 95% confidence intervals.

**Table 2:** Nonequilibrium work in the absence and presence of ERY in the exit tunnel.

*W*_pull_ (kcal/mol)	ErmCL	H-NS
Without ERY	305.0 ± 24.0	507.3 ± 20.1
With ERY	2009.6 ± 122.7	1267.8 ± 106.4

Error bars represent 95% confidence intervals.

### In the presence of ERY, ErmCL is harder to translocate through the tunnel than H-NS

**Figure 2:**
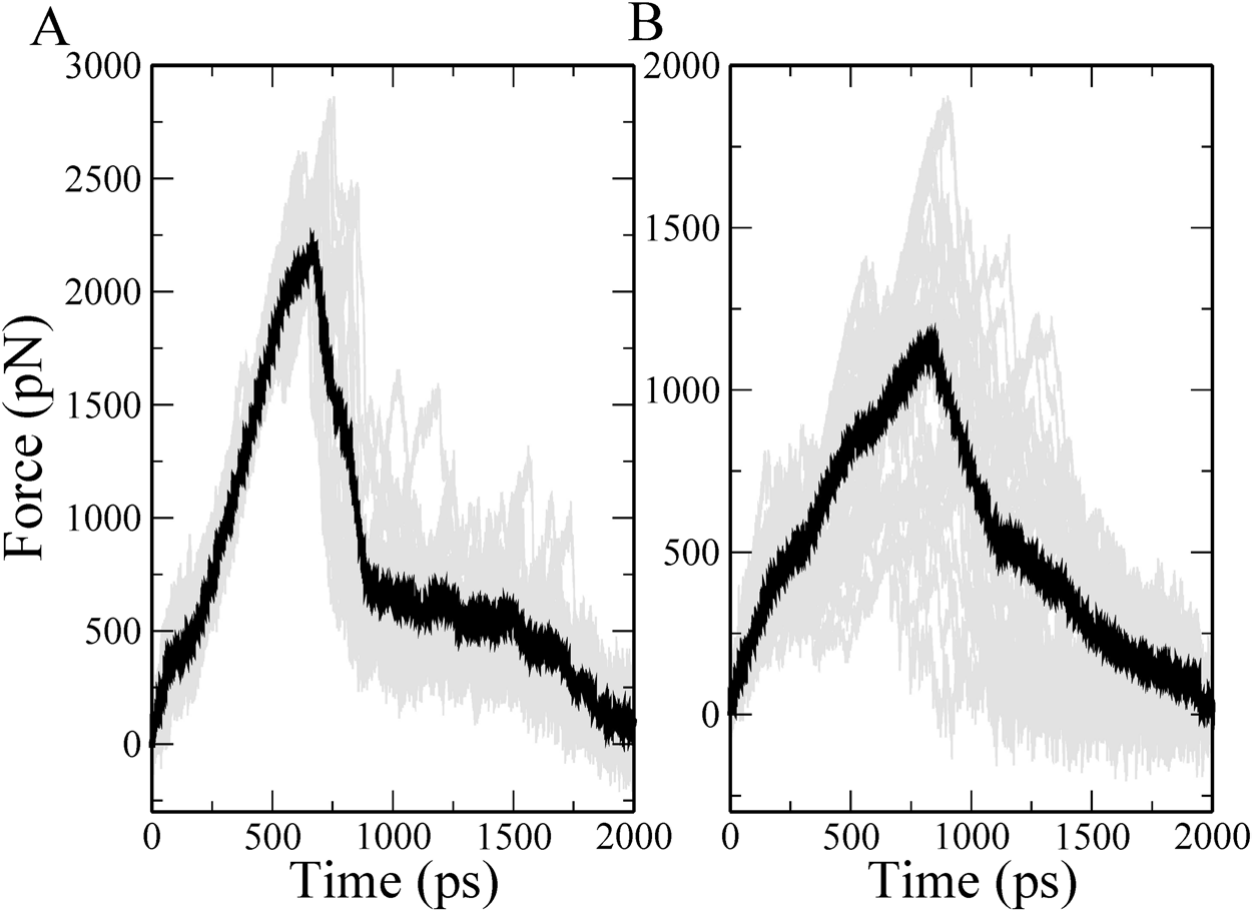
Pulling force versus time from the SMD simulations of (**A**) ErmCL and (**B**) H-NS with ERY bound in the exit tunnel. Black lines represent the average force across trajectories, while gray lines are the force traces for each individual trajectory.

The presence of ERY changes not only the magnitude of rupture force but also the shape of the force versus time profile. For ErmCL, the mean-maximum-rupture force is increased dramatically from 469 pN to 2,640 pN. Moreover, sharp peaks in the force profiles appear in the presence of ERY (Fig. 2). These results indicate that the presence of ERY in the exit tunnel impedes the movement of ErmCL through the tunnel. In the case of H-NS, the presence of ERY also increases its mean-maximum-rupture force from 599.9 to 1,394 pN, however, the rupture force is nearly half that of ErmCL. Additionally, the distribution of rupture forces is statistically different between the two peptides in the presence of ERY (Student’s t-test, $p$ = 6.43 × 10^−5^). Thus, ERY has a greater impact on the translocation of ErmCL than H-NS.

The non-equilibrium work of translocating these peptides also leads to similar conclusions (Table 2), as *W*_pull_ equals 2,009.6 (95% CI: [1886.9, 2132.3]) and 1,267.8 (95% CI: [1161.4, 1374.2]) kcal/mol for ErmCL and H-NS, respectively. The difference between the work distributions is statistically significant (Student’s t-test test, $p$ = 3.62 × 10^−8^). Thus, ErmCL needs more energy to translocate through the exit tunnel than H-NS. Because *W*_pull_ correlates with binding affinity^29^, ErmCL is expected to interact with ERY and the ribosome more strongly than H-NS. Therefore, the results from *F*_max_ and *W*_pull_ are consistent with the experiments showing that the translation of ErmCL is stalled by ERY, while H-NS can bypass it.

### Robustness of results to changes in pulling speed

To determine whether these conclusions are sensitive to the pulling speed used in the SMD simulations we have performed simulations in which the pulling speed is 5-fold larger, *i.e. v* = 25 m/s. Compared to the 5 m/s case, the rupture force and non-equilibrium work are higher for both ErmCL and H-NS. However, ErmCL still has a larger *F*_max_ and *W*_pull_ than H-NS (Figure S2 and Table S1) indicating that, consistent with the slower pulling speed, ErmCL is harder to pull through the tunnel than H-NS. Thus, our conclusions do not depend on pulling speed. In the rest of this study we report results in which v = 5 m/s in the SMD simulations.

### N-terminal residues bypassing ERY experience the largest forces

**Figure 3:**
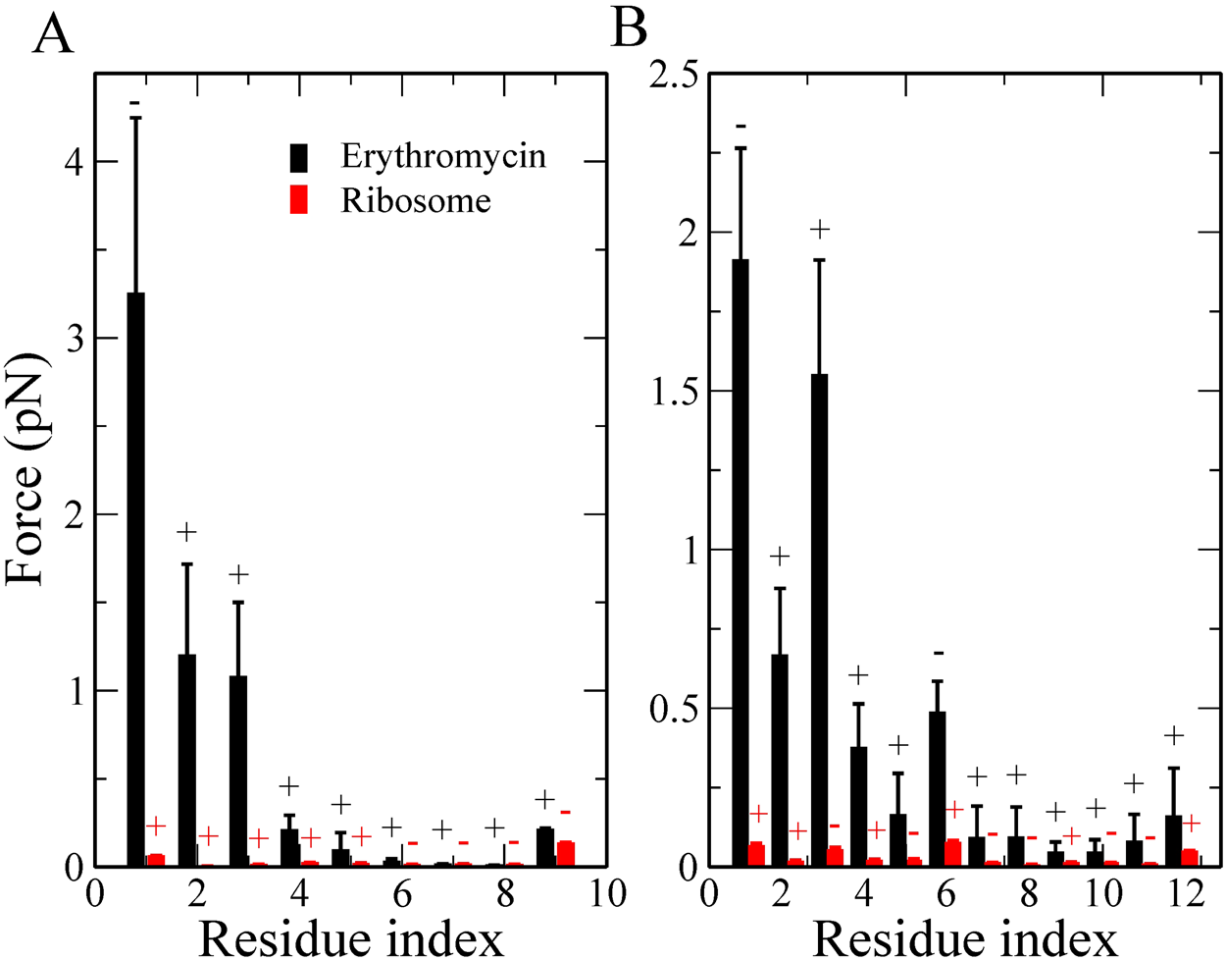
Average force per atom experienced by nascent chain residues at ${F}_{{\max }}$ for (**A**) ErmCL and (**B**) H-NS. Above each data point the ‘+/−’ signs indicate whether, on average, the force was attractive or repulsive, respectively. Error bars represent the 95% confidence interval about the mean.

To determine why ErmCL’s maximum pulling force is higher than that of H-NS’s we first calculated the average force acting on each residue along the nascent chain when *F*_max_ occurs in the individual trajectories, because at this point there is the greatest opposition to protein movement through the tunnel. We find that for both peptides, their N-terminal residues have larger forces acting on them than their C-terminal residues (Fig. 3). And that ErmCL’s first two N-terminal residues experience around a 70% greater force than in H-NS (compare Fig. 3A,B). This suggests that the differential behavior of ErmCL and H-NS in the presence of ERY originates at their N-terminal residues.

We hypothesized that structurally, the largest forces in the force profiles (Fig. 2) are generated when the peptides are passing through the constricted space formed between ERY and the exit tunnel wall. To test this, we determined the average distance between the center of mass (COM) of each nascent chain residue and ERY (Table 3) when *F*_max_ occurred in the individual trajectories. We find that for both peptides the N-terminal residue is closest to ERY at *F*_max_, and the C-terminal residues are increasingly further away. Thus, for both peptides, the largest forces arise as the N-terminal residues attempt to bypass ERY.

**Table 3:** Average distance (Å) between COMs of residues of nascent peptides and COM of ERY at *F*_max_.

Residue	ErmCL	H-NS
1	6.7 ± 0.6	10.2 ± 1.9
2	8.7 ± 0.3	9.3 ± 1.5
3	11.7 ± 0.4	9.9 ± 1.2
4	15.2 ± 0.5	11.1 ± 0.9
5	18.1 ± 0.5	13.9 ± 0.7
6	21.4 ± 0.5	15.8 ± 0.8
7	25.4 ± 0.5	18.9 ± 1.0
8	28.5 ± 0.5	21.3 ± 1.3
9	32.0 ± 0.5	24.1 ± 1.5
10	N/A	27.8 ± 1.8
11	N/A	31.0 ± 1.9
12	N/A	33.2 ± 2.0

Error bars represent 95% confidence intervals.

As follows from Fig. 3, the residues at the N-terminus appear to play a decisive role in ribosome stalling, but this contradicts experiments^9,11^ which have found that more C-terminal residues of ErmCL are important. In the next section we show that this discrepancy can be resolved by taking into account the fact that amino acids are added one at a time.

### Gradual addition of amino acids to a growing nascent peptide

The results presented thus far are from simulations in which the entire nascent peptide is present at all times during the simulation, which does not reflect what happens during protein synthesis. It is therefore possible that our results are affected by interactions between residues that would not normally be present at a given point during synthesis, and the rest of the system. To address this issue we more accurately modeled the synthesis process by only turning on non-bonded interactions of nascent peptide residues as they pass 4 Å’s into the exit tunnel, which is the approximate distance between successive amino acids.

**Figure 4:**
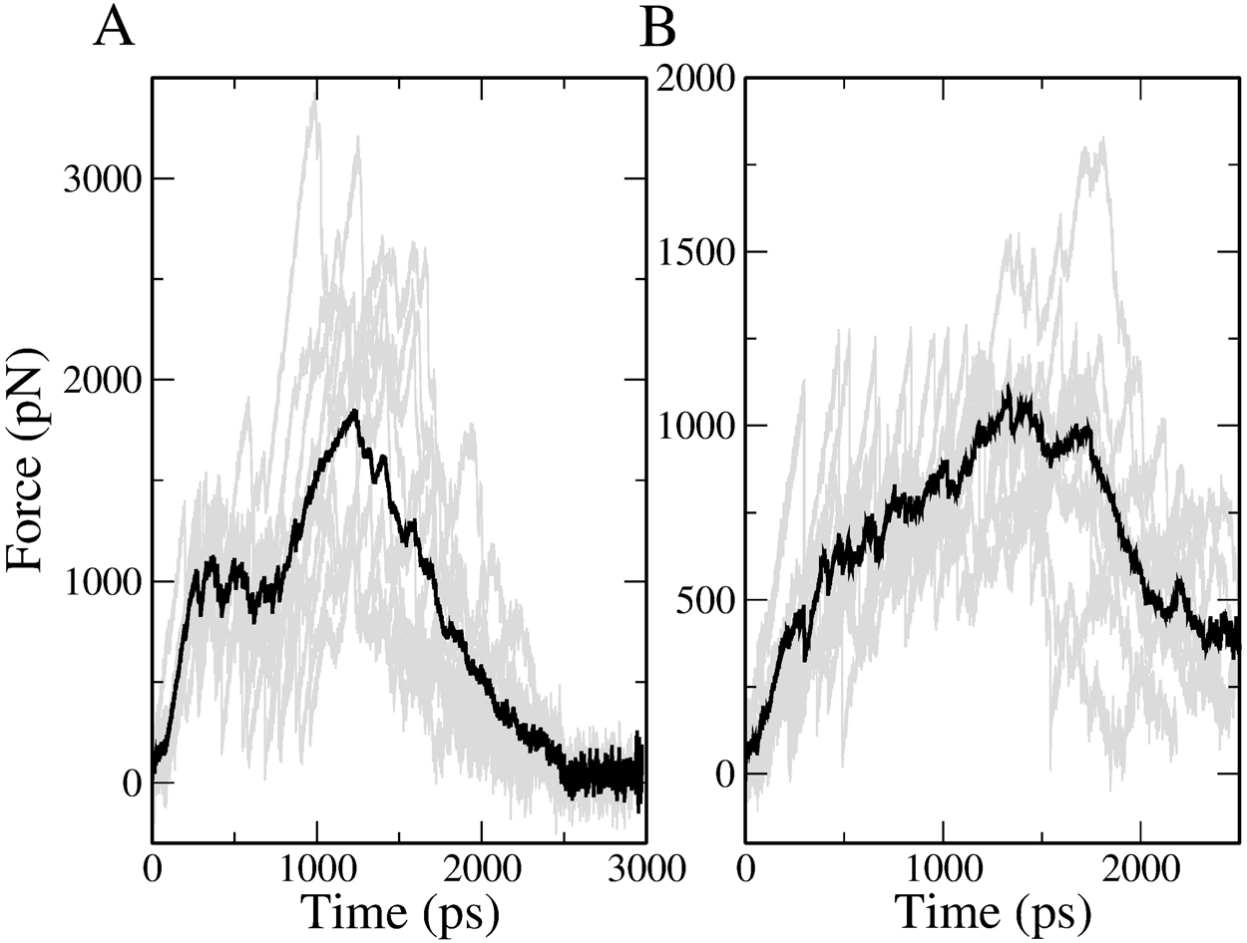
Pulling force versus time from the SMD simulations in which the non-bonded parameters are turned on gradually as nascent chain residues enter the exit tunnel for ErmCL (**A**) and H-NS (**B**) with ERY bound in the exit tunnel. Black lines represent the average force across trajectories, while gray lines are the force traces for each individual trajectory.

Using five different starting structures, ten simulation trajectories were simulated to obtain the force-time profiles shown in Fig. 4. The mean rupture forces of ErmCL and H-NS are 2,242 and 1,359 pN, respectively, indicating that ErmCL is harder to pull through the tunnel (Table 4). The non-equilibrium work also leads to a similar conclusion (Table 4). Thus, these results are in agreement with our earlier simulations.

**Table 4:** Nonequilibrium work and rupture force in the presence of ERY in the exit tunnel when the non-bonded parameters of residues of nascent peptides are turned on by sequence.

	ErmCL	H-NS
*W*_pull_ (kcal/mol)	1441.0 ± 286.8	957.0 ± 192.0
Rupture force (pN)	2242.4 ± 374.8	1358.6 ± 278.0

Error bars represent 95% confidence intervals.

**Figure 5:**
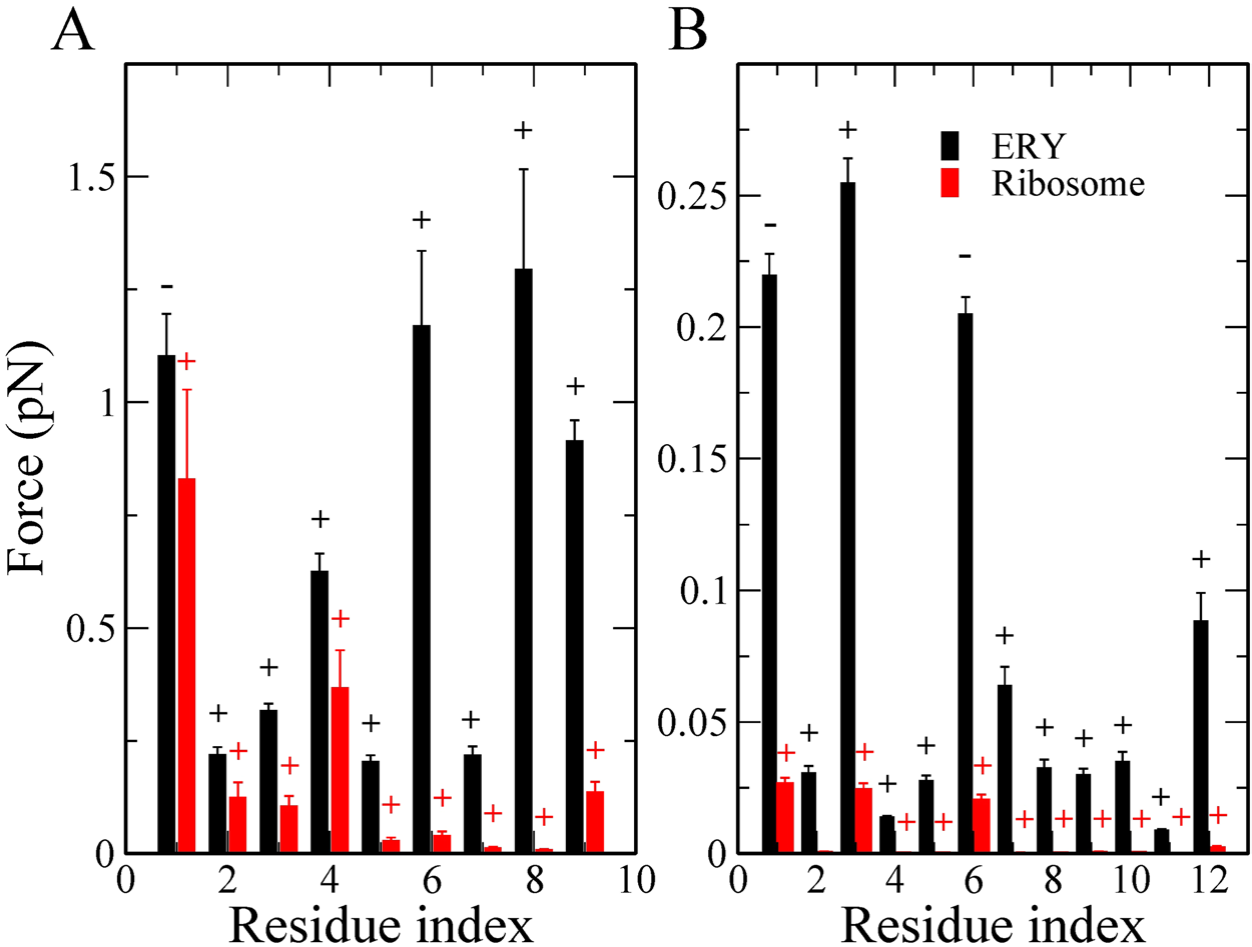
Average force per atom experienced by nascent chain residues at ${F}_{{\max }}$ for (**A**) ErmCL and (**B**) H-NS with non-bonded interactions are turned on one by one. The ‘+/−’ signs refer to attractive or repulsive forces, respectively. Error bars represent the 95% confidence interval about the mean.

As done before, we computed the force experienced at each residue position when the maximum force occurs. We find different results (Fig. 5) for these simulations. For ErmCL, residue 1 seems to substantially contribute to stalling but the strong interaction with ERY is compensated by interaction with ribosome. Residues 6 and 8 experience the largest force, indicating that the C-terminus needs more energy for passage through the tunnel than the N-terminus. Furthermore, residue 4 is closest to ERY at *F*_max_ (Table 5), and, within error, residues 3, 5 and 6 are also close to ERY suggesting that residues 3 through 6 are located near ERY when ErmCL attempts to bypass it. These findings are consistent with experiments^9,11^ showing that C-terminus of the ErmCL motif is critical for stalling and that residues 4–6 are located next to ERY. Our results also indicate that turning on non-bonded interactions residue-by-residue, which better mimics the protein synthesis process, allows the nascent peptide to better adapt to the presence of ERY during equilibration. In the earlier simulations the nascent peptide apparently did not have enough time to adapt to the presence of ERY, leading to a strong interaction of the N-terminal residues with ERY.

**Table 5:** Average distance (Å) between COMs of residues of nascent peptides and COM of ERY at *F*_max_ with non-bonded parameters turned on one by one residue.

Residue	ErmCL	H-NS
1	16.8 ± 4.2	19.1 ± 4.6
2	12.0 ± 3.2	16.4 ± 3.8
3	11.4 ± 2.8	15.5 ± 2.4
4	9.7 ± 2.1	15.0 ± 2.1
5	11.0 ± 2.8	14.4 ± 2.1
6	11.5 ± 2.7	17.7 ± 2.4
7	13.8 ± 3.5	18.5 ± 3.3
8	14.0 ± 3.3	19.4 ± 4.4
9	16.5 ± 3.4	21.0 ± 4.7
10	N/A	22.7 ± 5.0
11	N/A	25.6 ± 5.5
12	N/A	29.0 ± 5.1

Error bars represent 95% confidence intervals.

For H-NS, the N-terminus has stronger interactions with ERY than its C-terminus (Fig. 5), as found in the earlier simulations (Fig. 3). However, because H-NS has more time to adapt to the presence of ERY in this SMD setup, the rupture force becomes weaker. At *F*_max_ the COM distance between ERY and residue 5 is minimal (Table 5), but in contrast to ErmCL, the weak interaction between the C-terminus of H-NS and ERY (Fig. 5B, note the ~5-fold smaller interaction force in this figure compared to Fig. 5A) allows H-NS to bypass ERY easier. The importance of the C-terminus in H-NS’s ability to evade inhibition by ERY was also confirmed experimentally by Kannan *et al*.^14^. Therefore, in the analyses that follow, we focus on these simulations as they better mimic protein synthesis.

### The contribution of erythromycin to the forces the nascent chain experiences

**Figure 6:**
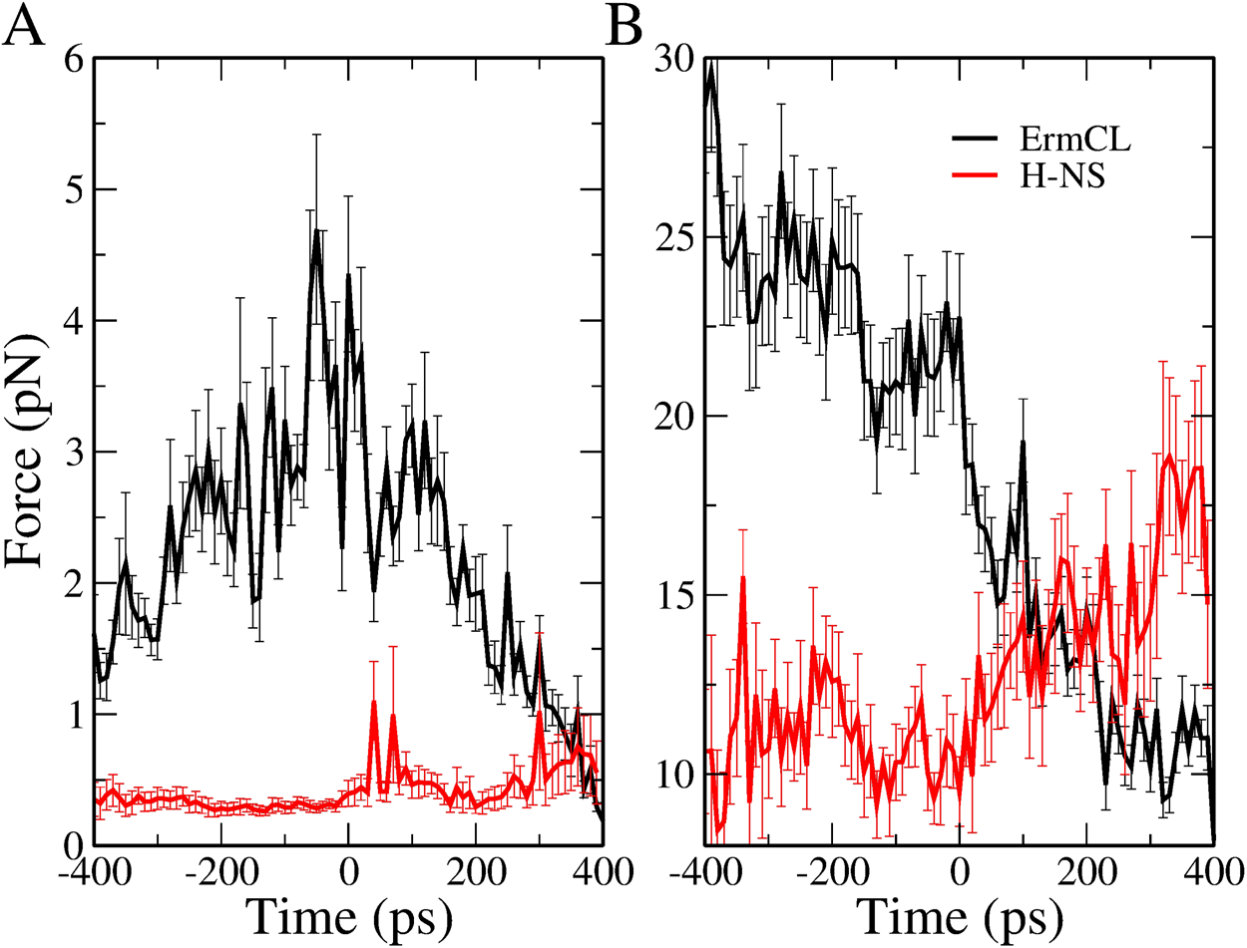
Average interaction force experienced by nascent chain atoms with ERY and the ribosome when we turned on non-bonded parameters one by one residue. (**A**) Average non-bonded interaction force, per nascent chain atom, between the nascent chain and Erythromycin. (**B**) Average non-bonded interaction force, per nascent chain atom, between the nascent chains and the ribosome. Error bars represent 95% confidence intervals. Line is to guide the eye and does not represent a model. Note well, the individual trajectory force traces were first aligned such that *F*_max_ occurs at time equal to zero, and then the average across trajectories was calculated.

To identify the molecular origins of the differential behavior of ErmCL and H-NS in our simulations we need to decompose the interactions of their residues with the ribosome and ERY. To determine the relative contribution of the ERY-Ribosome complex to the forces acting on ErmCL and H-NS, we calculated the forces arising from interactions of the nascent chain with the tunnel wall, and with ERY. These forces were calculated as a time series and normalized by the number of atoms in each component, with all of the trajectories post-processed to align the occurrence of their *F*_max_ value at $t$ = 0. We observe that when *F*_max_ occurs (at $t$ = 0), ErmCL experiences a higher interaction force with ERY than H-NS (Fig. 6A) as well as a 2-fold larger interaction force with the ribosome tunnel (Fig. 6B). This indicates that both ERY and the tunnel play a role in the force difference experienced by ErmCL and H-NS as they move through the exit tunnel, with the tunnel and ERY impinging more on ErmCL than H-NS.

### Electrostatic and dispersion interactions drive ERY’s differential impact on ErmCL

**Figure 7:**
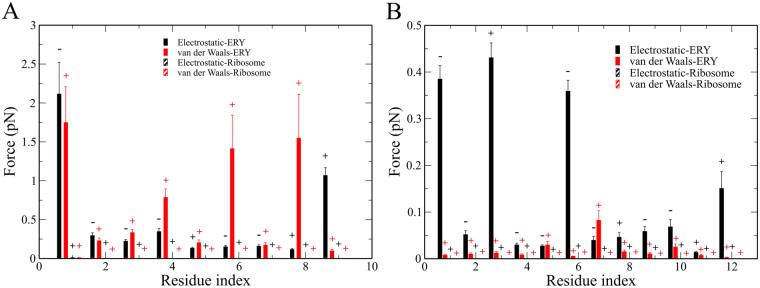
Force decomposition into electrostatic and van der Waals interactions between the nascent chains and ERY or the ribosome in the simulations in which non-bonded interactions are turned on as residues enter the exit tunnel. (**A**) Non-bonded interaction force at *F*_max_ between ErmCL’s residues ERY or the ribosome. (**B**) Non-bonded interaction force at *F*_max_ between H-NS’s residues and ERY or the ribosome. Above each data point the ‘+/−’ signs indicate, on average, whether the force was attractive or repulsive, respectively. Error bars represent 95% confidence intervals.

To determine which non-bonded interaction energies contribute the most to *F*_max_, we calculated the electrostatic and dispersion interaction force components between each nascent-chain residue and the tunnel wall and erythromycin at *F*_max_. We observe in both peptides the N-terminal residue exhibits electrostatic repulsion with ERY and attractive dispersion interactions (Fig. 7). The stronger interaction with ERY of the C-terminus compared to N-terminus of ErmCL come from dispersion interactions of residues 6 and 8 and the electrostatic interaction of residue 9 (Fig. 7A). The strong dispersion force between residue 6 and ERY implies that this residue is important in ribosome stalling. This result is consistent with Arenz *et al*.’s report that hydrophobic amino acids at position 6 maintain the stalling^11^. In the case of H-NS, the N-terminal interactions of residues 1, 3 and 6 with ERY are dominant (Fig. 7B). Furthermore, the non-bonded forces between ERY and ErmCL’s C-terminus are mainly attractive while in H-NS residues 1 and 6 exhibit repulsive electrostatic forces. This suggests that the differential impact that ERY has on ErmCL and H-NS’s motion through the tunnel arises from the larger electrostatic and dispersion forces their C-terminal residues experience.

## Discussion and Conclusions

We have performed two sets of SMD simulations. In the first set, protein synthesis was not been taken into account, *i.e*., the nascent peptide was treated as a preformed chain. In the second set each amino acid was added onto the peptide by gradually turning on its non-bonded interactions as it moved into the exit tunnel. We observed that in both sets of simulations it is harder to move ErmCL through the tunnel than H-NS (Figs 2 and 4). However, the interactions of individual nascent chain residues with ERY are different between the two sets of simulations. In the first set, the differential impact of ERY on ErmCL compared to H-NS arises in part from stronger intermolecular interactions between the N-terminal residues of ErmCL and ERY (Fig. 3), at which point the N-terminal nascent-chain residues are sandwiched between ERY and the ribosome exit tunnel (Table 4). In the second set of simulations, however, residues that were more C-terminal (residues 3 through 6) in ErmCL experienced the largest forces and therefore are more likely to contribute stalling. The interaction forces between ErmCL and ERY and the ribosome were about two-fold higher than the interaction forces of H-NS with these components (Fig. 6). Decomposing the pair-wise interactions, we observed that when the maximum force occurs, ErmCL experiences larger electrostatic and dispersion interaction forces with ERY than H-NS does (Fig. 7). Thus, ERY opposes the movement of both peptides through the tunnel, but the interactions with more C-terminal residues of ErmCL lead to even greater opposition than H-NS.

Other sequence features might also contribute to the differential impact of ERY. In ErmCL, residues 6I, 8 V and 9I of ErmCL are highly hydrophobic (with hydropathy indices for V and I of 4.5 and 4.2, respectively^30^) suggesting hydrophobic interactions might be important to ERY-driven ribosome stalling. Moreover, the average per-residue hydropathy index of ErmCL (25.2/9 = 2.8) is larger compared to H-NS (20.3/12 = 1.7). This suggests the possibility that the higher the hydrophobicity of the nascent peptide the greater the likelihood of arrest by ERY. This notion is supported by experimental data^9^ showing that the mutation F7A in ErmCL, which reduces the hydropathy index by 1.0 (hydropathy index of F and A is equal 2.8 and 1.8, respectively), promotes translocation of ErmCL through the tunnel exit.

Another sequence difference that might be important is that H-NS contains a positively charged residue K at position 6 that experiences a repulsive interaction with ERY which also carries a positive charge +1e (Fig. 5). This repulsive electrostatic interaction might facilitate H-NS bypassing of ERY. Thus, a nascent peptide containing residues that have a charge opposite to that of the macrolide might be more likely to be arrested. Taken together, the results and implications of this study could be tested by making mutations in the nascent chain that alter its hydrophobic or charge characteristics at residue positions that experience the largest force in our simulations.

In these simulations we applied a pulling force to the N-terminus of the nascent chain. In contrast, a transient pushing force may be experienced on the C-terminus of the nascent peptide as the A-site tRNA moves from the classical (A/A) to hybrid (P/A) to translocated (P/P) state. Once the tRNA has translocated to the P-site there should be little to no pushing force present (as the C-terminus is no longer moving), and the nascent chain can relax under the new conditions. Thus, provided quasi-equilibrium conditions are achieved under these conditions, and in the simulation conditions, the two situations should yield similar results. While the SMD simulations are clearly out-of-equilibrium, the robustness of our conclusions to varying the pulling speed suggests our results are independent of the irreversible pulling force.

Other molecular mechanisms have been identified or suggested to be the cause of ERY’s differential impact, such as the perturbed conformation of A76 in the P-site, which can lead to a decrease in translation rate^31^, or the reorientation of A2062 and A2503 that might allosterically affect the A-site crevice nucleotides thereby preventing peptide bond formation^12,31^. However, the mechanism we have identified is not mutually exclusive with these other mechanisms. The delay of nascent peptide movement past ERY, which our results suggest occurs, could change the PTC configuration due to a pile up of residues near the PTC.

Limitations of this study include the perennial issue of the accuracy of the all-atom force field employed and the rapid translocation of the peptide through the exit tunnel in the simulations. The all-atom force field we used is among one of the most accurate and successful in terms of ribosome simulations^13,31–34^. The rapid translocation of the peptide was necessary so as to obtain results in a realistic time frame. It is possible that the forces experienced by the peptide could change as the translocation speed is decreased. However, we have shown that even if this is the case, the qualitative differences we observed between ErmCL and H-NS (larger forces for ErmCL as compared to H-NS) are likely to persist. Thus, while the exact numbers may change, the trends and conclusions are not likely to.

More generally, this study illustrates the utility of using Steered Molecular Dynamics simulations to understand the mechanisms by which an antibiotic can act on a biological process. The 100-fold speed up of SMD simulations over conventional MD methods suggests that it may be possible to utilize a similar approach to screen potential antibiotics for their ability to inhibit protein synthesis.

## Electronic supplementary material

Supporting information
