# Supplementary material for: Erythromycin leads to differential protein expression through differences in electrostatic and dispersion interactions with nascent proteins
Source: Sci Rep. 2018 Apr 24;8:6460. doi: 10.1038/s41598-018-24344-9 (PMC5915450; doi:10.1038/s41598-018-24344-9)
Supplement: Supplementary file 1 — Supporting information [file 41598_2018_24344_MOESM1_ESM.docx]

**Erythromycin leads to differential protein expression through differences in electrostatic and dispersion interactions with nascent proteins**

Nguyen Hoang Linh^1^, Pham Dang Lan^1^, Edward P. O’Brien*^2^ and Mai Suan Li*^1,3^

*^1^Institute for Computational Sciences and Technology, Ho Chi Minh City, Vietnam*

*^2^Department of Chemistry, Pennsylvania State University, University Park, Pennsylvania 16802, USA*

*^3^Institute of Physics, Polish Academy of Sciences, Al. Lotnikow 32/46, 02-668 Warsaw, Poland*

**Supporting Information**


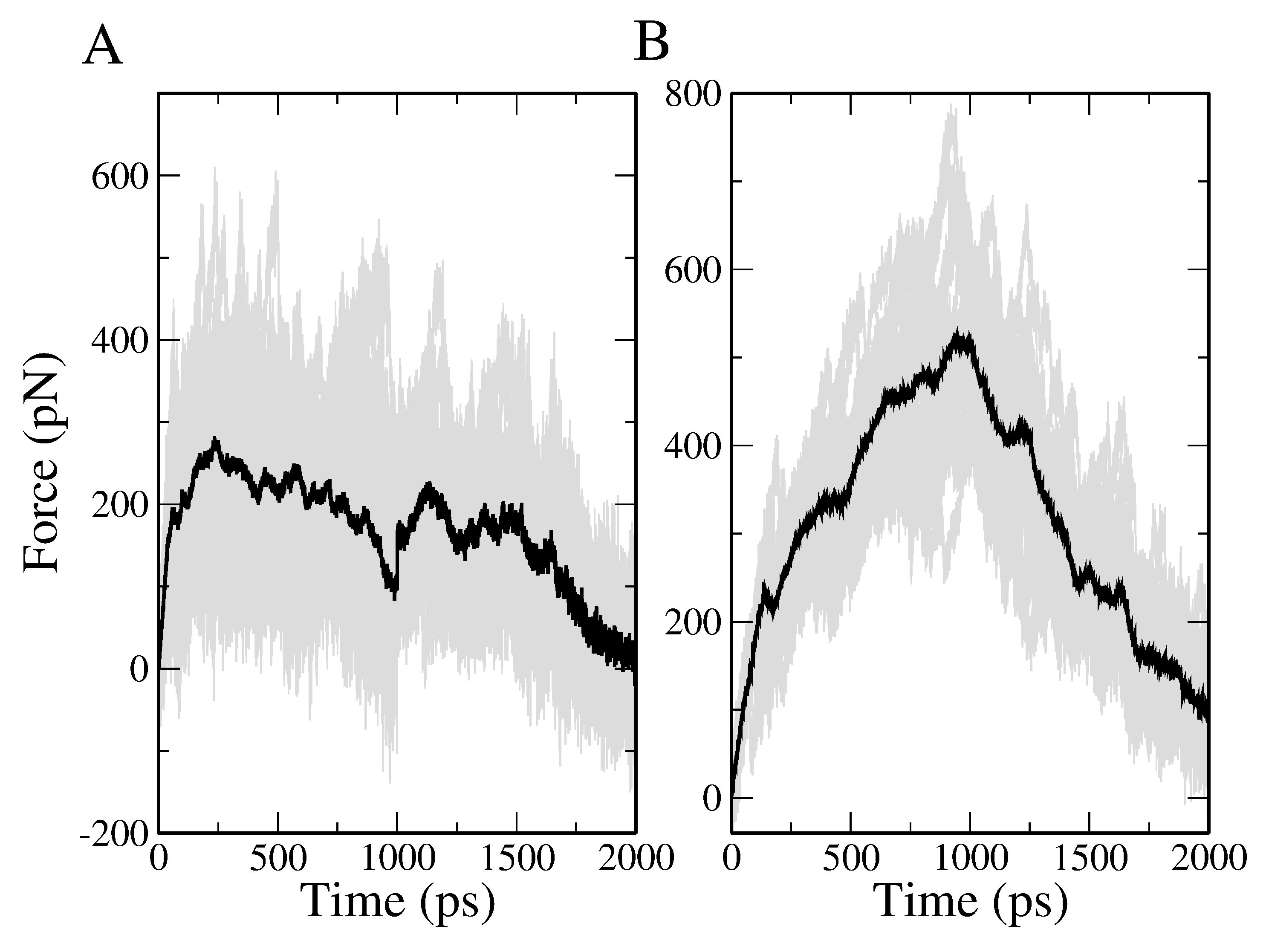
 **Figure S1.** **Pulling force versus time from the SMD simulations of (A)** ErmCL and **(B)** H-NS without ERY bound in the exit tunnel. Black lines represent average forces, gray lines are the traces of each individual trajectory.


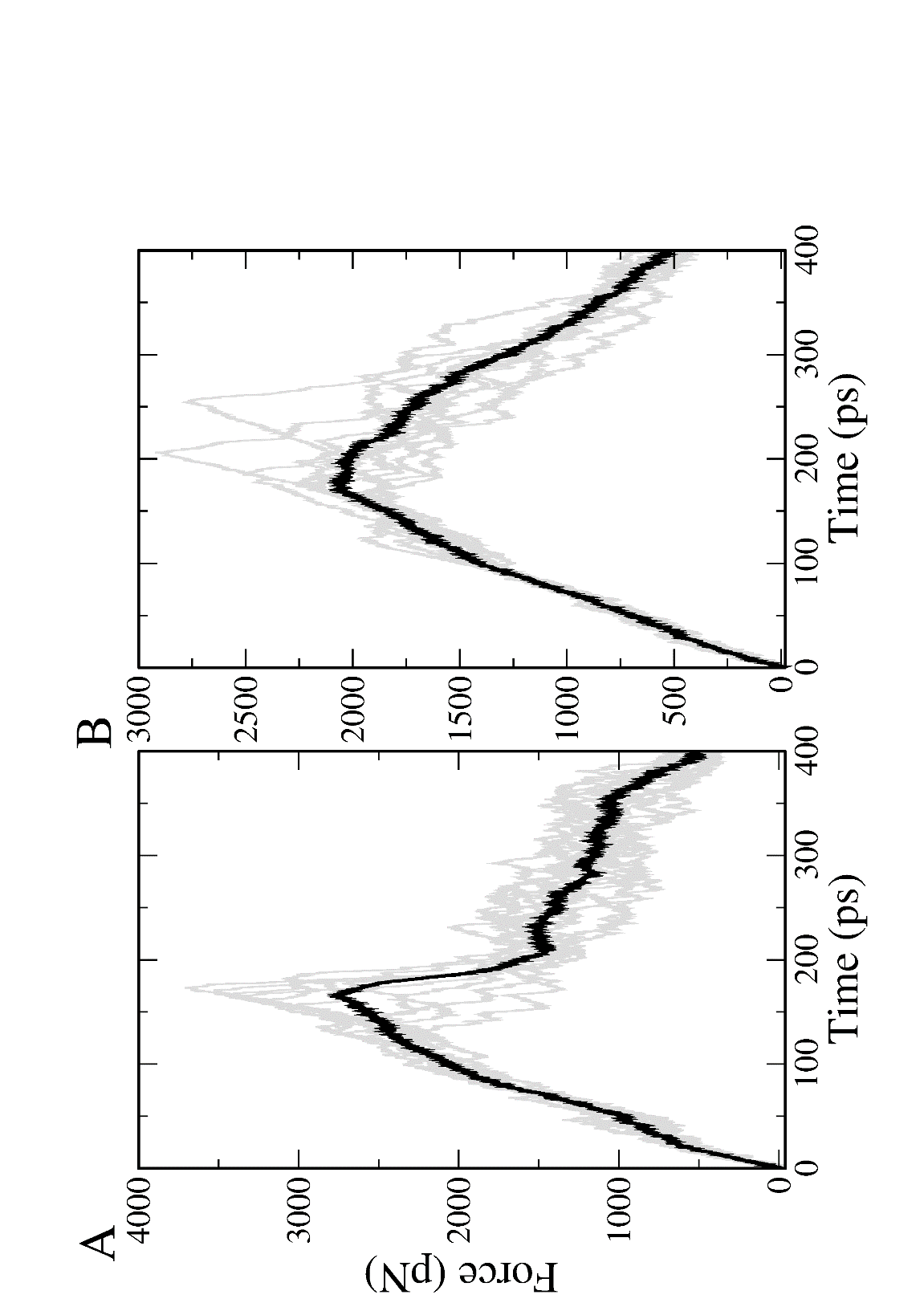


**Figure S2:** **Pulling force versus time from the SMD simulations with pulling velocity 25 m/s** of **(A)** ErmCL and **(B)** H-NS with ERY bound in the exit tunnel. Black lines represent the average force across trajectories, while gray lines are the force traces for each individual trajectory.

**Table S1**: Rupture force and work in the presence of ERY in the exit tunnel with pulling velocity 25 m/s. Error bars represent 95% confidence intervals.

|  | ErmCL | H-NS |
| --- | --- | --- |
| Rupture force (pN) | 3034.1 ± 289.1 | 2276.5 ± 209.2 |
| Pulling work (kcal/mol) | 2055.6 ± 64.4 | 1811.7 ± 95.3 |
